# Novel In Situ-Cross-Linked Electrospun Gelatin/Hydroxyapatite Nonwoven Scaffolds Prove Suitable for Periodontal Tissue Engineering

**DOI:** 10.3390/pharmaceutics14061286

**Published:** 2022-06-16

**Authors:** Martin Philipp Dieterle, Thorsten Steinberg, Pascal Tomakidi, Jiri Nohava, Kirstin Vach, Simon Daniel Schulz, Elmar Hellwig, Susanne Proksch

**Affiliations:** 1 Division of Oral Biotechnology, Center for Dental Medicine, Medical Center—University of Freiburg, Faculty of Medicine, University of Freiburg, Hugstetter Str. 55, 79110 Freiburg, Germany; martin.dieterle@uniklinik-freiburg.de (M.P.D.); pascal.tomakidi@uniklinik-freiburg.de (P.T.); simonds@web.de (S.D.S.); 2 Anton Paar TriTec SA, 2035 Corcelles, Switzerland; jiri.nohava@anton-paar.com; 3 Institute of Medical Biometry and Statistics, Medical Center—University of Freiburg, Faculty of Medicine, Albert-Ludwigs-University of Freiburg, 79104 Freiburg, Germany; kv@imbi.uni-freiburg.de; 4 Department of Operative Dentistry and Periodontology, Centre for Dental Medicine Medical Center—University of Freiburg, Faculty of Medicine, Albert-Ludwigs-University of Freiburg, 79106 Freiburg, Germany; elmar.hellwig@uniklinik-freiburg.de (E.H.); susanne.proksch@gmail.com (S.P.)

**Keywords:** periodontal guided tissue regeneration, mesenchymal stem cells, periodontal ligament, tissue engineering, regenerative medicine, biocompatible materials, gelatin, hydroxyapatites, periodontitis

## Abstract

Periodontal diseases affect millions of people worldwide and can result in tooth loss. Regenerative treatment options for clinical use are thus needed. We aimed at developing new nonwoven-based scaffolds for periodontal tissue engineering. Nonwovens of 16% gelatin/5% hydroxyapatite were produced by electrospinning and in situ glyoxal cross-linking. In a subset of scaffolds, additional porosity was incorporated via extractable polyethylene glycol fibers. Cell colonization and penetration by human mesenchymal stem cells (hMSCs), periodontal ligament fibroblasts (PDLFs), or cocultures of both were visualized by scanning electron microscopy and 4′,6-diamidin-2-phenylindole (DAPI) staining. Metabolic activity was assessed via Alamar Blue^®^ staining. Cell type and differentiation were analyzed by immunocytochemical staining of Oct4, osteopontin, and periostin. The electrospun nonwovens were efficiently populated by both hMSCs and PDLFs, while scaffolds with additional porosity harbored significantly more cells. The metabolic activity was higher for cocultures of hMSCs and PDLFs, or for PDLF-seeded scaffolds. Periostin and osteopontin expression was more pronounced in cocultures of hMSCs and PDLFs, whereas Oct4 staining was limited to hMSCs. These novel in situ-cross-linked electrospun nonwoven scaffolds allow for efficient adhesion and survival of hMSCs and PDLFs. Coordinated expression of differentiation markers was observed, which rendered this platform an interesting candidate for periodontal tissue engineering.

## 1. Introduction

The periodontium is a complex anatomical region within the oral cavity. It comprises different, histologically defined tissues, i.e., the gingiva, the periodontal ligament (PDL), cementum, and the alveolar bone. Gingival fibroblasts (GFs) and gingival keratinocytes (GKs) are the main constituents of the gingiva, while the PDL, among other tissues, harbors specialized fibroblasts called periodontal ligament fibroblasts (PDLFs) and periodontal ligament stem cells (PDLSCs) [1,2,3,4]. The cementum is populated by cementoblasts, and the alveolar bone consists of osteoblasts, osteoclasts, mesenchymal stem cells (MSCs), and an organic as well as an inorganic matrix [5,6,7,8,9]. Resulting from this variety of cell types and extracellular constituents and their mutual interaction, the developmental histogenesis and homeostasis of the periodontium are only incompletely understood in terms of the exact spatiotemporal molecular processes [9,10,11,12,13]. The periodontium can be severely destroyed by many oral diseases, including inflammatory processes such as gingivitis and periodontitis, and this lack of knowledge makes it difficult to adequately address these tissue defects from a therapeutic point of view. Since millions of people worldwide are affected by these debilitating diseases, their effective treatment is both a medical and an economic challenge [14,15,16,17,18].

Although innovative strategies such as guided bone regeneration [19,20], guided tissue regeneration [21,22], and stem cell-based therapies [23,24] have been intensely investigated in the context of periodontal tissue engineering, the clinical results of such translational approaches are still unsatisfactory [25,26]. Even highly sophisticated technologies such as biomaterial-supported cell-sheet transfer of periodontal cells into oral defect sites have not reached the clinic yet [27,28]. Thus, the current standard treatment options for severe periodontitis still rely on antimicrobial chemotherapeutics as well as periodontal debridement [29]. Consequently, novel approaches are urgently needed that (*i*) allow reproducible in vitro study of intercellular interactions of different periodontal cell types and (*ii*) offer a perspective for future periodontal tissue engineering in humans.

Against this background, polymer-based, biodegradable biomaterials have proven suitable as scaffolds that allow the in vitro modeling of complex biological processes such as soft and hard tissue regeneration, which are both important in the context of periodontal tissue engineering [30,31,32]. Various nontoxic natural and synthetic polymers, including chitosan [33], alginate [34], collagen/gelatin [35], and polylactic [36] and polyglycolic acid [37], have been adapted for similar applications and imitate natural extracellular matrices (ECMs) [38,39]. Among them, gelatin is a natural origin protein obtained by acidic and alkaline processing of collagen type I, the main protein component of the skin, bones, and connective tissue of animals. In this context, gelatin has been proven as biocompatible material that is not cytotoxic and has low immunogenicity compared with that of collagen. This material has been generally approved as safe by the Food and Drug Administration (FDA) [40]) and widely used in vitro and in vivo [41]. All of the aforementioned polymers are all easily and cost-efficiently processed by a fabrication method called electrospinning. In this method, polymer solutions are automatically extruded from syringes and become electrostatically accelerated towards a collector via high voltage. Subsequently, the solvent evaporates, which results in the deposition of nonwoven fibers. Through variation of the production parameters, properties such as fiber diameter, porosity, and material thickness can be reproducibly tailored to the specific experimental needs [42]. Multifunctional composite materials that consist of different components can be fabricated reliably at the nanometer scale [43,44,45,46]. Because of its many advantages, electrospinning has already been experimentally applied to bone [47,48], cartilage [49,50], tendon/ligament [51,52], skin [53,54], and cardiovascular [55,56] tissue engineering, as well as for wound dressings [57,58,59]. To date, however, no FDA-approved electrospun regenerative tissue engineering biomaterial is available commercially [42].

Nonetheless, electrospinning appears to be an optimal method for fabricating and optimizing a novel polymer-based nonwoven scaffold for periodontal tissue engineering. Such a scaffold should account for the specific needs of each cell type in the periodontium and allow for cellular adhesion, spreading, and proliferation. Its biomechanical properties, i.e., the spacing of cell adhesion points and the stiffness/elastic modulus (Young’s modulus), should be permissive for supporting and maintaining the proper differentiation of the cells of interest [58,59,60,61,62,63,64]. In previous studies, we showed that a gelatin/polycaprolactone-based nonwoven was suitable for establishing cocultures of GKs and GFs [31], proper stratification of keratinocytes [30,32], and substantial in vivo periodontal tissue engineering in a minipig soft tissue dehiscence model [31]. This proved the overall applicability of the method in the periodontal context, especially by supporting the epithelial/mesenchymal interface necessary for soft tissue regeneration applications of the oral cavity and other clinical biomaterial-based applications [60,61]. However, the in vitro reconstitution of a model harboring PDLFs and human MSCs (hMSCs) has, to the best of our knowledge, never been described so far.

hMSCs have gained increasing attention in the field of biomedicine over the last three decades [7,62,63,64]. Because of their developmental potency, hMSCs can differentiate into various cell types, including fibroblasts, adipocytes, chondrocytes, and osteoblasts [65,66]. The latter are especially interesting in the context of periodontal tissue engineering, since bone resorption and destruction is a common problem in periodontitis that is difficult to address therapeutically. Thus, hMSC-based periodontal tissue engineering strategies have been developed that have aimed at the in situ differentiation of hMSCs into osteoblasts for alveolar bone regeneration [11]. hMSCs can be readily isolated from adult donors and therefore offer the possibility of autologous, cell-based, regenerative therapies without immunological complications [67,68]. Exemplarily, scaffold-free clumps of MSCs and their self-synthesized ECM were investigated in a rat calvaria defect model and shown to induce bone regeneration [69]. hMSCs support osteogenesis through the secretion of soluble factors such as vascular endothelial growth factor (VEGF) and thereby contribute to alveolar bone regeneration through diverse mechanisms [70]. Consequently, hMSCs are an important source and mediator of hard tissue formation in the periodontium. The influence of hMSCs on PDL cells and vice versa is, however, only poorly understood. Experimental setups with cocultures of various periodontal cell types have suggested that intercellular interactions are key in determining tissue architecture and the induction or maintenance of the cellular phenotype [71]. Thus, interactive cocultures of PDLFs and hMSCs constitute an important biological interface of soft tissue cells with an inherent potential for hard tissue formation or induction. To investigate the interdependence of PDLFs and hMSCs in the periodontal context therefore represents an important challenge in current research.

The aim of this proof-of-principle study was to develop and in vitro validate an innovative, composite biomaterial suitable for periodontal tissue engineering. By using a mixture of gelatin and hydroxyapatite for electrospinning, the natural ECM of the periodontal ligament (gelatin) and the inorganic matrix of the periodontal hard tissues (hydroxyapatite, HA) was imitated. The scaffold was aimed at serving the biological needs of both PDLFs and hMSCs, i.e., allowing the adhesion, spreading, proliferation, and differentiation of each monoculture as well as interactive cocultures of both cell types. Since it is known that HA used as blend in nanofiber scaffolds enhances osteogenic differentiation in preosteoblasts and mMSCs [72,73], we embedded HA nano-particles in our scaffolds during electrospinning processing. The incorporation of additional porosity in a subset of scaffolds led to further optimization of the cell compatibility of the nonwovens. Comparison of different culture conditions and analysis of biological parameters such as cell density, cell penetration, metabolic activity, and cellular differentiation validated the concept. The presented data are an important basis for further analysis of the biological needs of periodontal cells, tissue–tissue interactions in the periodontium, and the translation of nonwoven-based scaffolds into clinically applicable biomaterials for human periodontal tissue engineering.

## 2. Materials and Methods

**Fabrication of electrospun gelatin/hydroxyapatite scaffolds with (eGHA_ap_) and without additional porosity (eGHA):** Gelatin powder (EMPROVE^®^, pharmaceutical grade; Merck KGAA, Darmstadt, Germany) was solubilized in a mixture of acetic acid (Merck KGAA), ethyl acetate (Sigma-Aldrich, Munich, Germany), and water in a ratio of 5:3:2 [31]. The gelatin concentration used for eGHA scaffold generation was 16% (*w/v*), and 5% (*w*/*v*) hydroxyapatite nanopowder < 200 nm particle size (Sigma-Aldrich, Munich, Germany) was added. For in situ cross-linking, glyoxal (40% glyoxal solution; Sigma-Aldrich) at a concentration of 1.63 mmol glyoxal cross-linker per gram gelatin was applied and mixed with the gelatin/hydroxyapatite solution at room temperature (RT). Then, the gelatin/hydroxyapatite/glyoxal mixture was filled into syringes of 17 Gauge and mounted in a syringe pump (type: KDS100 or KDS101; KD Scientific, Hollisten, MA, USA). The spinning process was performed with flow rates of 10–30 mL/h for each scaffold for 2 h, which yielded a substrate area of 50 cm^2^ with a gelatin/hydroxyapatite areal density of 0.02 g/cm^2^ and a fiber diameter of 528 ± 17 nm, as validated in a previous study [30,74]. Two high voltage power generators (Heinzinger LNC 30000-2 neg. and LNC 30000-2 pos., Rosenheim, Germany) were used to establish an electric field between the cannulae and the collector for “jet-stream formation”. The field forces were between 1.9 and 3 kV/cm. To generate scaffolds with additional porosity, one third of the syringes for electrospinning were filled with polyethylene glycol (PEG 200, Sigma-Aldrich) 16 g/100 mL (*w*/*v*), yielding water-soluble fibers that were then washed out with ethanol/water at RT. Subsequent temperature treatment (80 °C; 2 h) resulted in additional cross-linking of the gelatin/hydroxyapatite fibers with glyoxal. Before in vitro preclinical validation experiments, eGHA scaffolds were wetted in Aq_dest_ for 2 × 10 min at RT. eGHA and eGHA_ap_ were sterilized with 70% ethanol for 2 × 20 min and washed for 10 min in sterile phosphate-buffered saline (PBS, Life Technologies, Darmstadt, Germany) at RT.

**Biomechanical characterization of the gelatin/hydroxyapatite scaffolds**: The mechanical properties of the nonwovens were characterized in cooperation with Anton Paar TriTec SA, Buchs, Switzerland. Generally, the elastic modulus/Young´s modulus of randomly oriented nonwoven scaffolds is difficult to measure. Therefore, a Bioindenter™ UNHT**^3^** Bio (Anton Paar, Graz, Austria) with a large cylindrical indenter (diameter 1 mm) was used to average the biomechanical properties of a relatively large area of the nonwovens. The samples were fixed on the bottom of a Petri dish cover using two-compound epoxy (Carl Roth, Karlsruhe, Germany). The mixture was smeared on the bottom of the Petri dish cover in a thin layer and cured for 2 min in order not to penetrate into the nonwoven fiber mesh. A small piece of each nonwoven (approximately 15 × 15 mm) was then carefully placed on the epoxy layer and lightly pressed with the help of an aluminum block to achieve flat surfaces. This mounting was air-dried for at least 1 h to achieve full cure of the epoxy. Tap water was then poured onto the nonwovens; this was followed by a soaking period of 1 h. Subsequently, the actual indentation experiments were performed. All indentation tests were executed in force control mode to a maximum load of 0.1 mN with loading and unloading rates of 0.6 mN/min and a 15-second hold period at maximum force. The elastic modulus E was calculated using Equation (1):

$$E=\frac{S}{D}$$

where S is the slope of the unloading curve (see Supplementary Figure S1) and D is the diameter of the cylindrical indenter. At least five indentations spaced 1.5 mm apart were performed on each sample. An average value and standard deviation were calculated for each sample using the method initially described by Oliver and Pharr [75]. Exemplary measured curves of one eGHA and one eGHA_ap_ scaffold are shown in Supplementary Figures S2 and S3, respectively.

**Cell culture:** All experiments were conducted in accordance with the guidelines of the World Medical Association Declaration of Helsinki and approved by the Committee of Ethics of the Medical Faculty of Albert-Ludwigs-University Freiburg, Germany (EK-153/15). Human bone marrow-derived mesenchymal stem cells (hMSC) were obtained from pelvic bone aspirate remnants of a healthy patient undergoing hMSC-based sinus floor augmentation (technically supported by the bone marrow aspiration pack, Harvest Technologies Corp., Plymouth, MA, USA). The plastic-adherent cells were cultivated in NH expansion medium supplemented with CytoMix (both Miltenyi Biotec, Bergisch Gladbach, Germany), passaged up to 2-4 times (P2–4), and stored in liquid nitrogen until usage. Furthermore, the hMSC-inherent clonogenicity, multilineage potential, and surface marker expression were assessed as reported previously [74]. Primary human periodontal ligament fibroblasts (PDLFs) were obtained from the healthy periodontal ligament tissues of noncarious human premolar teeth extracted from two donors for orthodontic reasons. The periodontal ligament was scraped off from the middle third of the tooth roots. Specimens of the periodontal ligaments were rinsed with 10% iodine (B. Braun, Melsungen, Germany), thoroughly washed, and minced, and tissue fragments were plated as explants in minimum essential medium (MEM) alpha (Life Technologies, Darmstadt, Germany), supplemented with 10% fetal bovine serum (Biochrom, Berlin, Germany) and 1% GlutaMax™ (Life Technologies). The medium was exchanged every 2–3 days (d) until cell outgrowth. Upon confluency, cells were trypsinized (0.25% trypsin/EDTA, Anprotec, Bruckberg, Germany) and expanded by splitting. Passages P2–4 of the primary PDLFs were used for further experiments.

**Establishment of hMSC and PDLF monocultures and interactive hMSC/PDLF cocultures on eGHA_ap_/eGHA:** eGHA and eGHA_ap_ scaffolds were fabricated as described above, cut into circular pieces with a diameter of 15 mm, and subsequently sterilized with 70% ethanol (see above), washed with PBS (see above), and placed into 24-well plates (Becton Dickinson, Heidelberg, Germany). The nonwovens were equilibrated overnight in DMEM (Life Technologies) supplemented with 50 µg/mL kanamycin (Sigma-Aldrich). The next day, the DMEM was removed. For monoculture experiments, 1 x 10^5^ hMSCs (in supplemented NH proliferation medium, see above) or PDLFs (in MEMalpha medium, see above) were suspended in a small amount of the corresponding media, seeded on only one side of each scaffold, and incubated at 37 °C and 5% CO_2_ for 3, 7, 10, 14, or 21 d. For coculture experiments, 1 x 10^5^ hMSCs per nonwoven were suspended in a small amount of supplemented NH proliferation medium (see above) and seeded on only one side of the scaffold. After 1 h of preincubation at 37 °C and 5% CO_2_, the wells were filled up to approximately 1 mL with supplemented NH medium (see above). The cells on the scaffold were incubated for another 2 d. For PDLF cultivation on the other side of the scaffold, the scaffold was flipped upside down in the well and seeded with 1 × 10^5^ PDLFs per nonwoven in a small amount of MEMalpha medium (see above). After another preincubation step of 1 h, the wells were filled up to 1 mL with supplemented MEMalpha medium (see above) and incubated at 37 °C and 5% CO_2_ for 3, 7, 10, 14, or 21 d.

**Scanning electron microscopy (SEM):** To assess cellular adhesion to eGHA and eGHA_ap_ scaffolds, cell-seeded devices were fixed with 3.8% paraformaldehyde (PFA) (Sigma-Aldrich) at d 10 and d 21 and stored at +4 °C in PFA until further usage. After rinsing with PBS, specimens were dehydrated via an ascending alcohol series (ranging from 30 to 100% ethanol three times each for 20 min at RT). Critical point drying was performed (CPD 030 Critical Point Dryer, Bal-Tec AG, Balzers, Liechtenstein), and the samples were immediately sputter coated with a 10 nm sheet of gold/palladium for 60 s at 60 mA (SCD 050, Balzers, Liechtenstein). The scaffolds were examined using an LEO 435 VP scanning electron microscope (Zeiss, Oberkochen, Germany).

**DNA and protein stains:** Specimens were fixed in ice-cold 70% ethanol, dehydrated in an ascending ethanol series (80, 90, and 100% ethanol for 1 h each), embedded in paraffin (Engelbrecht Medizin und Labortechnik, Edermünde, Germany), and cut into 5 µm sections using a Leica microtome (Leica RM2255, Wetzlar, Germany). For cell number and migration distance visualization, samples were deparaffinized, and nuclei were stained with 300 nM 4**′**,6-diamidin-2-phenylindole (DAPI, Carl Roth) for 1 min and mounted with Fluoromount G™ (Biozol, Eching, Germany). Nuclei were visualized with a BZ-9000 fluorescence microscope (Keyence, Neu-Isenburg, Germany) and analyzed (interactive cocultures vs. hMSCs only vs. PDLFs only, each with and without additional porosity (eGHA or eGHA_ap_) and at different points in time (d 3, 7, 10, 14, or 21); see Supplementary Tables S1 and S2). The cell count per area (cells/1000 µm^2^) was quantified for each section using the BZ II Analyzer software (Keyence). Cell migration distance/maximum cell penetration into the nonwoven (µm) was measured and averaged along *n* = 3 perpendiculars to the corresponding surface tangents per section.

Cell differentiation was assessed on d 21 by immunohistochemical staining of tissue sections, which were dewaxed and incubated in DIVA antigen retrieval solution (Biocare Medical, Concord, CA, USA) at 60 °C overnight. The staining was performed using rabbit (rb) anti-human osteopontin (1:100 in PBS with 2% bovine serum albumin (BSA: Sigma Aldrich; antibody: Abcam, Cambridge, UK)), rb anti-human periostin (1:100 in PBS with 2% BSA, (antibody: Abcam)), mouse (ms) anti-human Oct4 (1:100 in PBS with 2% BSA, (antibody: Sigma Aldrich)), and ms anti-human vimentin (1:200 in PBS with 2% BSA, (antibody: Abcam)) antibodies. After blocking of endogenous peroxidase with 3% H_2_O_2_ (Sigma Aldrich) and unspecific binding sites (5% BSA with 0.25% Triton X-100; Sigma Aldrich) in normal horse serum for 60 min at RT, sections were exposed to the aforementioned primary anti-human osteopontin, periostin, or Oct4 antibodies overnight at 4 °C. The sections were then washed with PBS and incubated with a biotinylated anti-mouse or anti-rabbit antibody for 1 h at RT (Vector Laboratories, Burlingame, CA, USA) and then exposed to preformed avidin–biotin peroxidase complexes (ABC horseradish peroxidase [HRP] Kit solution; Vectastain, Vector Laboratories). Antigen visualization was performed by adding a freshly prepared substrate solution containing 3,5-diaminobenzidine (DAB) following the manufacturer’s instructions (Abcam, Cambridge, UK). Next, all sections were incubated with the vimentin antibody for 1 h at RT. This was followed by an incubation with an HRP-labelled anti-mouse secondary antibody (LI-COR Biosciences, Bad Homburg, Germany) for 30 min and FastGreen 0.1% (Carl Roth) for 1 min. After a final washing step, slides were briefly counterstained with hematoxylin (Sigma Aldrich), washed with tap water, dehydrated, and mounted with TechnoVit 7200 (Heraeus Kulzer, Wehrheim, Germany). Negative controls without primary antibodies were routinely included for each sample, each antibody, and every staining procedure. This staining protocol yielded hematoxylin- and vimentin-stained sections with additional immunohistochemical staining of either osteopontin, periostin, or Oct4.

**Metabolic activity assay:** The metabolic activity of the cells seeded on eGHA or eGHA_ap_ scaffolds was measured using the resazurin/Alamar Blue^®^ assay (Bio-Rad, Munich, Germany). Briefly, the culture medium of *n* = 3 independent samples per group (interactive hMSC/PDLF cocultures vs. hMSCs only vs. PDLFs only vs. cell-free scaffolds, each cultivated on either eGHA or eGHA_ap_) and point in time (d 1, 3, 7, 10, 14, or 21) was replaced by a culture medium containing 10% (*w/v*) Alamar Blue^®^ reagent. After incubation for 3 h at 37 °C and 5% CO_2_, triplicate samples of the supernatant were analyzed by measuring fluorescence intensity according to the manufacturer’s instructions (570 nm excitation and 630 nm emission wavelength) in an infinite-M microplate reader (Tecan, Männedorf, Switzerland). The relative amount of Alamar Blue^®^ reduction in the samples was calculated by using a 100% reduced Alamar Blue^®^ control as a reference. Blanks and negative controls were routinely included in each run.

**Statistics**: For each day and each condition, a linear mixed regression model using the restricted maximal likelihood (REML) method was fitted to evaluate the influence on each outcome of interest (total cell count/section area, migration distance, and Alamar Blue^®^ reduction rate). All conditions were pairwise compared with the corresponding baseline values using the Wilcoxon signed-rank test. All *p*-values were corrected for multiple testing according to the Bonferroni or Scheffé method, depending on the situation. Results were considered statistically significant if *p* < 0.05 and highly statistically significant if *p* < 0.01. The calculations were performed with the statistical software STATA 17.0 (StataCorp LLC., College Station, TX, USA).

## 3. Results

### 3.1. Electrospinning Allowed the Fabrication of Mechanically-Defined Gelatin/Hydroxyapatite Nonwovens

In this study, we aimed at generating a biocompatible nonwoven scaffold, which (i) supports the adhesion and growth of two cell types found within the periodontium, i.e., hMSCs and PDLFs, and (ii) allows for distinct expression of differentiation markers in these cells.

**Figure 1:**
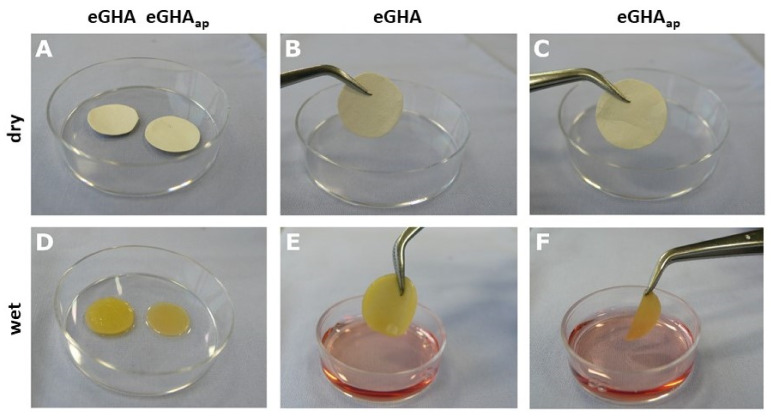
Photographs of electrospun gelatin/hydroxyapatite scaffolds with (eGHA_ap_) and without additional porosity (eGHA). The scaffolds were punched in circles after drying (**A**–**C**) and subsequently rewetted with culture medium (**D**–**F**). (**A**–**C**) If dry, there was no remarkable macroscopic difference between eGHA and eGHA_ap_ (A, left: eGHA, right: eGHA_ap_) apart from the slightly papery appearance of eGHA_ap_ (**C**) when compared with that of eGHA (**B**). (**D**–**E**) Following wetting and moisture expansion, eGHA appeared clearly thicker (**D**, left) and inherently more stable (**E**), while eGHA_ap_ collapsed if taken with forceps (**F**) but remained in shape and easily unfolded if laid down (**D**, right).

Due to adaptable and cost-effective manufacturing, nonwoven-based scaffolds were generated by electrospinning. Gelatin (denatured/hydrolyzed collagen) was chosen as a basic material for the scaffolds, which is also a main component of the periodontal ligament and the organic matrix of the mineralized periodontal tissues [30]. To additionally simulate the mineralized matrix, hydroxyapatite was incorporated into the electrospinning solution. The gelatin concentration used for scaffold generation was 16% [*w*/*v*], while hydroxyapatite was added at 5% [*w*/*v*]. In situ cross-linking was enabled by the addition of glyoxal, finally yielding the electrospun gelatin/hydroxyapatite (eGHA) scaffolds. To test for the most cell-favorable conditions, some of the model substrates were fabricated with additional porosity, which imitate a porous, mineralized matrix. This additionally enhances the permeability for nutrients and was achieved by incorporating water-extractable polyethylene glycol fibers into the nonwovens. These fibers were washed out subsequently. The latter scaffolds with additional porosity are correspondingly designated as eGHA_ap_. Figure 1 exemplarily shows photographs of the nonwovens before being seeded with cells. After drying (Figure 1A–C), there was no striking macroscopic difference between eGHA (Figure 1A(left),B) and eGHA_ap_ (Figure 1A(right),C). However, upon wetting with culture medium (Figure 1D–F), eGHA nonwovens were apparently thicker and mechanically more resistant (Figure 1D(left),E) than eGHA_ap_ (Figure 1D(right),F). This observation is relevant for assessing the clinical usability of the eGHA and eGHA_ap_ scaffolds because a sufficient dimensional stability is required for proper handling in the clinical context.

The elastic modulus/Young´s modulus of biomaterials is an important parameter that determines cell behavior. Different tissues possess differing mechanical properties that are fine-tuned to support the adhesion and differentiation of the resident cells. The exemplary quantitative mechanical characterization of the scaffolds via Bioindenter™ measurements yielded Young´s moduli in the range of 6.4 kPa for both eGHA and eGHA_ap_ (see Supplementary Figures S1–S3). Since previous studies showed that biomaterial scaffolds with a Young´s modulus in the low kPa range of 3.2 kPa can support gingival tissue formation, it was assumed that these material parameters might be suitable to allow the adhesion and spreading of hMSCs and PDLFs.

### 3.2. Electrospun Gelatin/Hydroxyapatite Scaffolds with and without Additional Porosity Were Efficiently and Densely Populated by Both hMSCs and PDLFs

**Figure 2:**
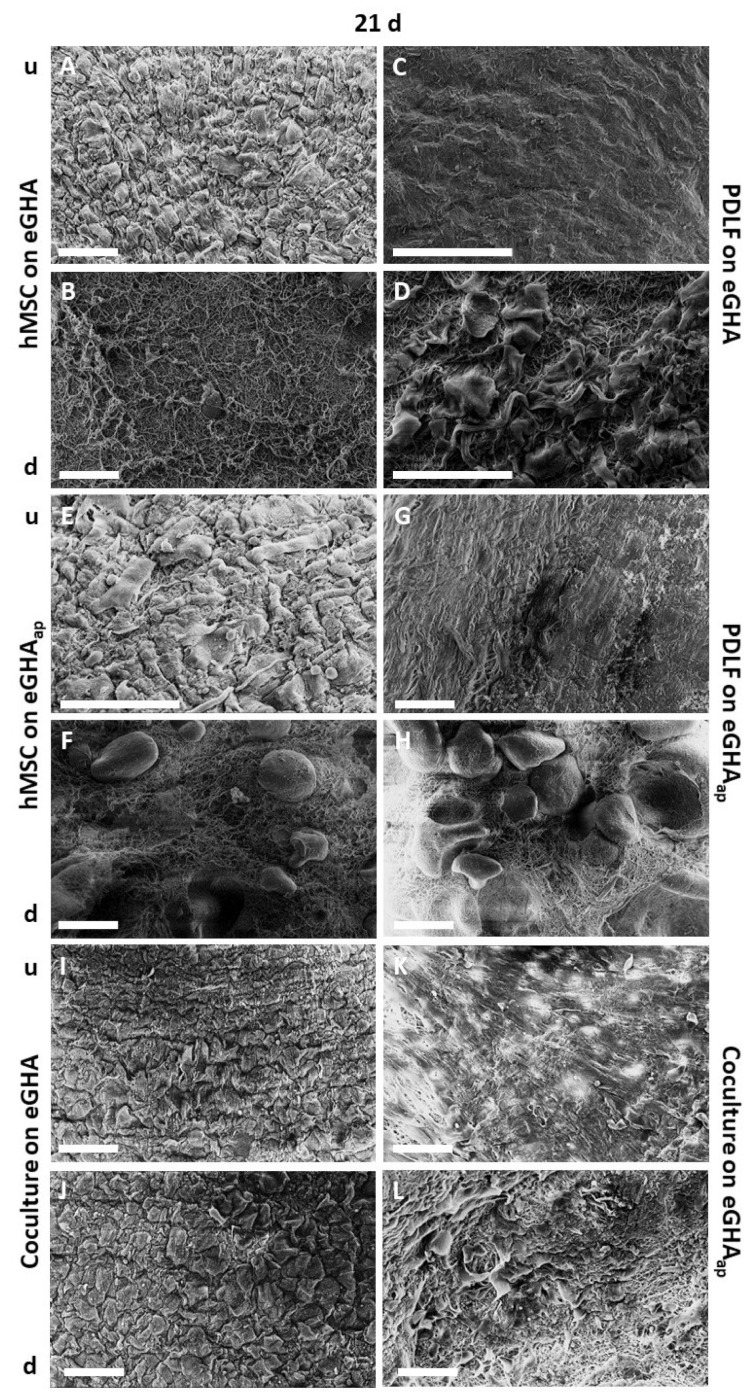
Scanning electron micrographs of eGHA/eGHA_ap_ scaffolds after 21 d. Illustrated is always the surface of the scaffolds, indicating the different morphologies of the respective cell types under study which from time to time reveal the nanofibers on the underside of the scaffolds (**B**,**D**,**F**,**H**,**J**,**L**). hMSCs (**A**,**B**,**E**,**F**), PDLFs (**C**,**D**,**G**,**H**), or cocultures of both (**I**–**L**) were seeded on either eGHA (**A**–**D**,**I**,**J**) or eGHA_ap_ scaffolds (**E**–**H**,**K**,**L**) and prepared for scanning electron microscopy (SEM) analysis after 21 d. The top sides (u) of the monocultures (**A**,**C**,**E**,**G**) were densely populated with either hMSCs or PDLFs, while the bottom sides (d) (**B**,**D**,**F**,**H**) illustrated the geometric configurations of the nonwovens and were barely populated by cells, as expected. In the cocultures, (u) were populated by hMSCs (**I**,**K**) and (d) with PDLFs (**J**,**L**). Details are given in the main text. All scaffolds, irrespective of the presence of additional porosity, were densely covered with the indicated cells, proving the overall suitability of the eGHA/eGHA_ap_ nonwovens for the adhesion and spreading of periodontal fibroblasts and mesenchymal stem cells. The cell morphologies could be described as polygonal or spindle-like. Scale bars represent 100 µm.

It was then tested whether the scaffolds could be populated by hMSCs, PDLFs, or interactive cocultures of both cell types. As described above, 1 × 10^5^ cells of each type were transferred to the nonwovens. After 10 d or 21 d for hMSCs and PDLFs, respectively, the samples were prepared for scanning electron microscopy (SEM) analysis. The corresponding SEM micrographs are depicted in Supplementary Figure S4 (analysis after 10 d) and Figure 2 (analysis after 21 d). Figure 2A represents the hMSC-populated eGHA scaffold from the top side (u), where the cells were placed, and Figure 2B represents it from the bottom side (d), which faced the culture dish. By analogy, Figure 2C,D shows the PDLF-populated eGHA scaffolds. hMSC-populated eGHA_ap_ nonwovens are depicted in Figure 2E,F; PDLF-harboring eGHA_ap_ specimens are shown in Figure 2G,H. Figure 2I–L shows SEM pictures of interactive cocultures of hMSCs and PDLFs (Figure 2I,J: eGHA; Figure 2K,L: eGHA_ap_); hMSCs are shown in Figure 2I),K and PDLFs are depicted in Figure 2J,L. The same system is used in Supplementary Figure S4.

All scaffolds were densely populated by hMSCs, PDLFs, or interactive cocultures at both points in time. The cells exhibited polygonal or slightly spindle-like morphologies, with some cells harboring protrusions. Abnormal or unexpected morphologies were absent. Cell demarcations were especially visible on hMSC-populated scaffolds but nearly absent for PDLFs, which indicated a higher degree of differentiation of the latter cell type. Qualitative visual assessment of the SEM micrographs pointed towards an increased cell density after 21 d compared with that after 10 d. In this experimental setup, no obvious differences in cell density or morphology between eGHA and eGHA_ap_ scaffolds could be detected. The downsides of the SEM micrographs of the hMSC- and PDLF-populated samples also illustrate the geometrical configurations of the nonwovens. Interestingly, coculture formation was supported by the nonwovens, proving that these biomaterials, with approximate stiffnesses of 6.4 kPa, enabled simultaneous adhesion of both periodontal cell types.

### 3.3. Additional Porosity of eGHA_ap_ Scaffolds Favored Cell Adhesion and Proliferation

As shown by SEM analysis, the surfaces of the scaffolds supported cell adhesion and enabled the growth of dense cell populations of both hMSCs and PDLFs. In the context of periodontal diseases, tissue defects are often of considerable size and depth. This means that periodontal tissue engineering needs to address the volume reduction induced by these lesions. For optimal restitution of tissues with the help of biomaterials, it is therefore desirable that cells can migrate into the scaffolds. This supports structural and functional regeneration of the defect by enabling, e.g., 3D cell interactions and connective tissue resynthesis.

To assess the density of the cultivated cell populations and to test whether cells can penetrate the scaffolds, hMSCs, PDLFs, and cocultures of both cell types were cultivated on eGHA or eGHA_ap_ scaffolds, as described above. After 3 d, 7 d, 10 d, 14 d, or 21 d, the constructs were fixed with ethanol, dehydrated, embedded in paraffin, and cut into sections of approximately 5 µm. Subsequently, the sections were deparaffinized and incubated with the fluorescence dye 4′,6-diamidin-2-phenylindole (DAPI), which stains DNA.

**Figure 3:**
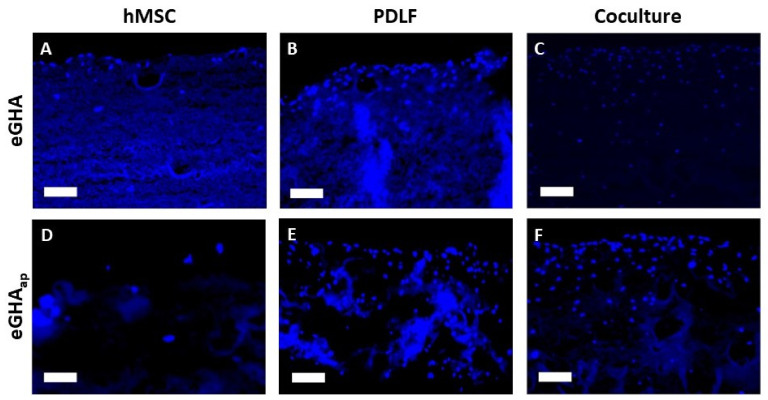
Representative cutouts of 4′,6-diamidin-2-phenylindol**e** (DAPI) stained sections from eGHA and eGHA_ap_ nonwovens populated with hMSCs, PDLFs, or cocultures after an incubation period of 21 d. The upsides of the monoculture scaffolds are oriented towards the top of the picture. For the cocultures (**C**,**F**), the margins of the PDLF-populated downsides are shown. (**A**) hMSCs, (**B**) PDLFs, or (**C**) cocultures were grown on eGHA scaffolds. Cell nuclei are presented as blue dots, while parts of the nonwovens also show some background fluorescence. Accordingly, eGHA_ap_ samples populated with (**D**) hMSCs, (**E**) PDLFs, or (**F**) cocultures are presented, showing a tendency towards increased cell densities when compared to eGHA nonwovens. Scale bars represent 100 µm.

**Figure 4:**
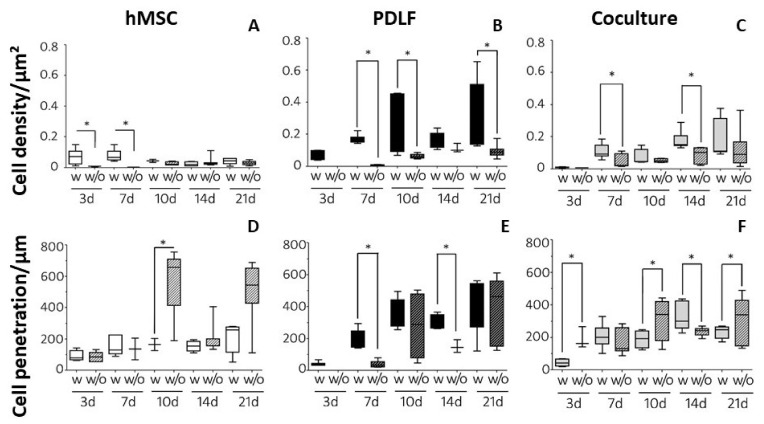
Statistical evaluation of the 4′,6-diamidin-2-phenylindol**e** (DAPI) stained sections after incubation for 3, 7, 10, 14, and 21 d. (**A**–**C**) Quantitative analysis of cell densities (cells/µm^2^) on nonwovens with (=eGHA_ap_ = w) or without (=eGHA = w/o) additional porosity populated by (**A**) hMSCs, (**B**) PDLFs, and (**C**) cocultures of both cell types. The boxplots represent the median values and interquartile ranges. The whiskers depict the 1.5-fold interquartile ranges. PDLFs on d 3 on eGHA could not be evaluated for technical reasons. (**D**,**E**) Quantitative analysis of the maximum cell penetration (µm) on nonwovens with (=eGHA_ap_ = w) or without (=eGHA = w/o) additional porosity populated by (**D**) hMSCs, (**E**) PDLFs, or (**F**) cocultures of both cell types. The boxplots represent the median values and interquartile ranges. The whiskers depict the 1.5-fold interquartile ranges. Numerical data for the graphs can be found in the Supplementary Tables S1 and S2. *: *p* < 0.05.

Representative regions from stained sections of all culture conditions after 21 d are shown in Figure 3. The cell densities (cells/µm^2^) were also evaluated quantitatively by systematically counting stained nuclei (three cutouts from three sections per condition and point in time each) and statistical analysis (Figure 4A–C, Supplementary Table S1).

Under almost all conditions, except for hMSCs on d 14, cell densities were higher on eGHA_ap_ than on eGHA (see Figure 3A–C vs. Figure 3D–F; Supplementary Table S1). This indicated that the additional porosity favored cellular adhesion and survival on the scaffolds. However, when analyzing the cocultures, cell types could not be distinguished via DAPI staining, meaning that the cell densities calculated for these experimental setups represented averages of hMSCs and PDLFs (Figure 3C,F, and Figure 4C). Notably, there was a general trend towards lower cell densities on hMSC-populated scaffolds than under the other conditions (Figure 4A vs. Figure 4B,C). For the PDLFs and cocultures, there was a clear tendency towards higher cell densities at later points in time (Figure 4B,C). This indirectly showed that cells could survive and proliferate on the scaffolds for a period of at least three weeks.

Separate statistical analysis of cell densities for each cell type, point in time, and scaffold revealed that there was no statistically significant difference in cell densities for hMSCs on either eGHA or eGHA_ap_ over time. Contrarily, for PDLFs on eGHA, cell densities were significantly different on d 10 vs. d 7, d 14 vs. d 7, and d 21 vs. d 7. For PDLFs on eGHA_ap_, statistically significant results were obtained for d 10 vs. d 3, d 21 vs. d 3, d 10 vs. d 7, d 21 vs. d 7, d 14 vs. d 10, and d 21 vs. d 14. As with hMSCs on eGHA, there were no statistically significant differences in the pairwise comparisons of points in time for cocultures on eGHA. However, for cocultures on eGHA_ap_, the comparisons of d 21 vs. d 3, d 10 vs. d 7, d 14 vs. d 7, d 21 vs. d 7, d 21 vs. d 10, and d 21 vs. d 14 were statistically significant. These data also underscored the favorable milieu provided by the additional porosity and supported the finding that PDLFs survived and proliferated more efficiently on the scaffolds.

Next, the influence of the scaffolds on cell densities was analyzed pairwise for each cell type and point in time (Figure 4A–C). As described above, cell densities were generally higher on eGHA_ap_ constructs. At d 3, there was a statistically significant difference in cell densities only between hMSCs on eGHA and those on eGHA_ap_ (Figure 4A). Interestingly, on d 7, the difference between the scaffolds was significant for hMSCs (Figure 4A), PDLFs (Figure 4B), and the cocultures (Figure 4C). However, the cell densities between eGHA and eGHA_ap_ at d 10 and d 21 were significantly only different for the PDLF-populated scaffolds (Figure 4B). On d 14, the cell densities were also significantly different in the coculture setting (Figure 4C).

A comparison of the cell densities for each cell type at each point in time, irrespective of the scaffold used, was performed next. Highly significant results were found on d 14 for PDLFs vs. hMSCs and cocultures vs. hMSCs. On d 21, similar results were detectable for PDLFs vs. hMSCs.

Taken together, these results proved that the culture time, the cell entity, and the scaffold all influenced the cell densities. eGHA_ap_ and longer cultivation times favored higher cell densities, as did using PDLFs or cocultures.

To further quantify the cellular behavior on the scaffolds, the maximum penetration depth was evaluated. This means that the maximum perpendicular distance of cells from the tangent touching the scaffolds´ surfaces was measured three times for each sample. The values were in the range of 37 to 580 µm (Supplementary Table S2). The results were analyzed statistically and are depicted in Figure 4D–F. For the monocultures, the side on which the cells were initially seeded was chosen as the reference for the tangent. In the coculture setting, the maximum penetration depth from the side on which the PDLFs were seeded was assessed. Interestingly, there was no statistically significant difference in the penetration depth among the cell types at d 3, 7, 10, 14, or 21 when analyzed independently of the scaffold used. The only exception was the comparison between the cocultures and hMSCs on d 14.

Analysis of the effect of the scaffold, i.e., eGHA versus eGHA_ap_, via pairwise comparison of each cell type at the indicated points in time revealed that there was a significant difference in the cell penetration depth of hMSCs on d 10, with greater penetration into the eGHA scaffolds (Figure 4D). The opposite was true for PDLFs on d 7 and d 14 (Figure 4E), where eGHA_ap_ scaffolds favored penetration. In the cocultures on d 3, d 10, and d 21 (Figure 4F), cells could migrate significantly deeper into the eGHA nonwovens. On d 14 (Figure 4F), however, they migrated significantly deeper into the eGHA_ap_ nonwovens.

Within each condition, i.e., with separate analysis for every cell type and scaffold, pairwise comparison of all points in time showed highly significant differences in penetration depths for hMSCs on eGHA on d 10 vs. d 3 and d 21 vs. d 3. For hMSCs on eGHA_ap_, no such highly significant results were found. PDLFs on eGHA differed highly significantly in penetration depths when comparing d 14 vs. d 7 and d 21 vs. d 7. The same cell type on eGHA_ap_ exhibited similar results for d 7 vs. d 3, d 10 vs. d 3, d 14 vs. d 3, and d 21 vs. d 3. In the coculture setting, no highly significant results were found on eGHA, but some were found on eGHA_ap_ for d 7 vs. d 3, d 14 vs. d 3, d 21 vs. d 3, and d 14 vs. d 7.

In summary, while eGHA_ap_ scaffolds supported higher cell densities, the data on the cell penetration depths showed that neither eGHA nor eGHA_ap_ nonwovens clearly favored cellular penetration. The maximum penetration depth varied considerably among the conditions, and there was no unambiguous trend for larger penetration depths over the course of the experiments. The data, however, did indicate that the cells were generally able to transmigrate the nonwovens, which is an important prerequisite for structural remodeling of defect sites in vivo.

### 3.4. Measure of Metabolic Activity of hMSCs and PDLFs Cultivated on Nonwoven Gelatin/Hydroxyapatite Scaffolds (eGHA) and Scaffolds with Additional Porosity (eGHA_a_)

**Figure 5:**
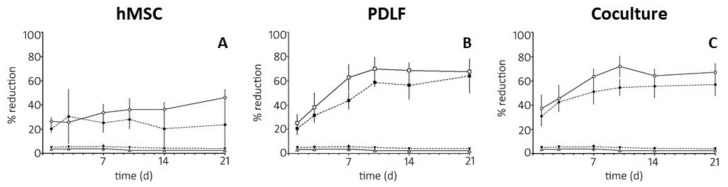
Line graphs representing the metabolic activities of hMSCs, PDLFs, or cocultures of both cell types on eGHA or eGHA_ap_. The resazurin/Alamar Blue^®^ activity assay was used to determine the reductive activity of the cells, which is an indirect measure for cell viability and metabolism. The indicated cells were incubated on the scaffolds for 1, 3, 7, 10, 14, and 21 d. Completely reduced Alamar Blue^®^ reagent was used as a positive control (=100%). eGHA (black triangles) and eGHA_ap_ (white triangles) without cells were used as negative controls for all experimental conditions. Mean metabolic activities and the corresponding standard deviations (SD) are depicted. The numerical data are presented in Supplementary Table S3. (**A**) Comparison of the reductive capacity of hMSCs grown on eGHA (black rhombs) and eGHA_ap_ (white rhombs). (**B**) Comparison of the reductive capacity of PDLFs grown on on eGHA (black squares) and eGHA_ap_ (white squares). (**C**) Comparison of the reductive capacity of interactive cocultures grown on eGHA (black circles) and eGHA_ap_ (white circles).

Qualitative and quantitative analysis of cell adhesion, proliferation, and penetration via DAPI staining revealed an advantage of eGHA_ap_ for total cell density but not for cell penetration. The mere presence of the cells, however, did not prove their metabolic integrity. The ability of cells to reduce substrates such as the phenoxazine dye resazurin (Alamar Blue^®^) is an important indicator of active metabolism and thus cell viability. Therefore, the Alamar Blue^®^ assay was used to assess the metabolic activity and cell viability of the hMSCs, PDLFs, and cocultures on eGHA and eGHA_ap_. As described in the Materials and Methods sections, the constructs were grown for 1, 3, 7, 10, 14, or 21 d and subsequently incubated with the Alamar Blue^®^ solution for 3 h. The relative metabolic activities for all conditions (100% = completely reduced Alamar Blue^®^ solution), which were derived from the percentage of reduced resazurin, were measured photometrically and are depicted in Figure 5 and Supplementary Table S3.

As can be seen in Figure 5A, metabolic activity was higher (except for d 3) when hMSCs were grown on eGHA_ap_ (white rhombs) than when they were grown on eGHA (black rhombs). This was in accordance with the increased cell densities on eGHA_ap_ scaffolds, as discussed in Section 3.3. Notably, there was a trend towards increased metabolic activities for the hMSC-populated eGHA_ap_ nonwovens with time, which was not the case for the eGHA constructs.

The overall relative metabolic activity of PDLFs on both eGHA (black squares) and eGHA_ap_ (white squares) was higher than that for hMSCs at later points in time (Figure 5B). Again, resazurin reduction was more pronounced in the cells on eGHA_ap_ scaffolds. Contrarily to the hMSCs, there was a clear tendency of increasing metabolic activity over time for PDLF-populated nonwovens. The results mirrored the cell densities on the corresponding scaffolds, as described in Section 3.3. The results supported the notion that PDLFs proliferated efficiently on the nonwovens. The metabolic activities of the cocultures were similar to those measured in the PDLF experiments (Figure 5C). The resazurin reduction was less pronounced on the eGHA (black circles) than on the eGHA_ap_ nonwovens (white circles). This was, again, in accordance with the cell densities on the corresponding scaffolds (see Section 3.3).

Statistical analyses of the cellular metabolic activities were also performed. When comparing the different scaffolds, i.e., eGHA versus eGHA_ap_, for each cell type and point in time separately, the differences in metabolic activity were statistically significant on d 1 for hMSCs. On d 3, there were no statistically significant results. However, on d 7, the scaffolds differed significantly for hMSCs, PDLFs, and the cocultures, whereas on d 10, they differed only for PDLFs and the cocultures. After 14 d and 21 d, statistical significance was reached only in the coculture setting and hMSCs, respectively.

Pairwise comparison of each cell type and scaffold for every point in time revealed no highly statistically significant results for hMSCs on eGHA or the empty controls (scaffolds without cells to assess baseline color change) on eGHA or eGHA_ap_. However, metabolic activity in hMSCs on eGHA_ap_ differed with high significance on d 21 vs. d 1, d 10 vs. d 3, d 14 vs. d 3, d 21 vs. d 3, and d 21 vs. d 7. For PDLFs on eGHA, all pairwise comparisons, except for d 14 vs. d 10, d 21 vs. d 10, and d 21 vs. d 14, were highly statistically significant. Similarly, PDLFs on eGHA_ap_ showed highly significantly different results in metabolic activities except for d 14 vs. d 7, d 21 vs. d 7, d 14 vs. d 10, d 21 vs. d 10, and d 21 vs. d 14. In the coculture setting, pairwise analysis of the time effect exhibited similar results for eGHA and eGHA_ap_. For eGHA scaffolds, all comparisons except for d 10 vs. d 1, d 10 vs. d 7, d 14 vs. d 7, d 21 vs. d 7, d 14 vs. d 10, d 21 vs. d 10, and d 21 vs. d 14 were highly significant; for cocultures on eGHA_ap_ nonwovens, the statistical analysis of the metabolic activity was nearly the same. Among the significant comparisons for cultures on eGHA scaffolds, only d 3 vs. d 1 was not highly significantly different on eGHA_ap_ scaffolds, whereas d 10 vs. d 1 was.

Taken together, the analysis of the metabolic activities of hMSCs, PDLFs, and cocultures on eGHA or eGHA_ap_ scaffolds underscored the biocompatibility of the nonwovens, since cells not only adhered but survived, were metabolically active, and proliferated for a substantial amount of time. For all cell types, the finding that eGHA_ap_ was superior to eGHA in terms of cell density was substantiated by higher metabolic activities on the nonwovens with additional porosity.

### 3.5. eGHA and eGHA_ap_ Scaffolds Allowed the Expression of the Differentiation Markers Oct4, Periostin, and Osteopontin

Apart from adhesion, proliferation, and metabolic activity, cellular differentiation is a key function for tissue homeostasis and integrity. Thus, biomaterials applied for regenerative purposes should permit or actively support the expression of differentiation markers in the cells they harbor. In this context, the gelatin/hydroxyapatite nonwovens were evaluated for characteristic biomarker expression of hMSCs and PDLFs.

**Figure 6:**
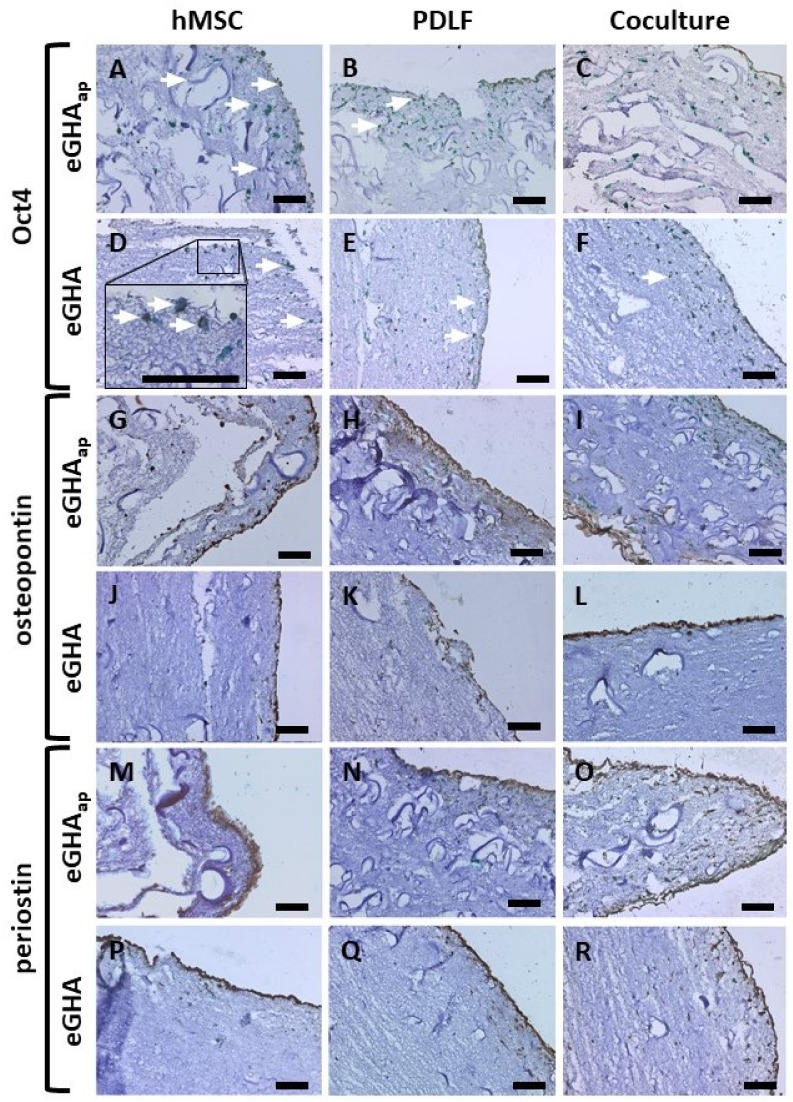
Immunohistochemical evaluation of eGHA and eGHA_ap_ scaffolds populated with hMSCs, PDLFs, and cocultures of both cell types. After an incubation period of 21 d, the constructs were fixed and incubated with primary antibodies against Oct4 (**A**–**F**), osteopontin (**G**–**L**), and periostin (**M**–**R**) and stained with the peroxidase-dependent 3,5-diaminobenzidine reaction (DAB; brown color). Subsequently, immunohistochemical staining for the mesenchymal cytoskeletal filament vimentin (HistoGreen staining; green color) was performed for all sections to detect all cells. Finally, hematoxylin counterstaining was applied. For each protein of interest, hMSCs (**A**,**D**,**G**,**J**,**M**,**P**), PDLFs (**B**,**E**,**H**,**K**,**N**,**Q**), and cocultures (**C**,**F**,**I**,**L**,**O**,**R**) grown on either eGHA_ap_ (**A**–**C**,**G**–**I**,**M**–**O**) or eGHA (**D**–**F**,**J**–**L**,**P**–**R**) were examined. The orientation of the representative pictures follows the same rules as in Figure 3. Staining results were evaluated visually and only qualitatively. (**A**,**D**) Oct4 was expressed mainly in hMSCs and detectable in only a few PDLFs (**B**,**E**). The inset in (**D**) represents a higher magnification of the section, illustrating the nuclear localization of Oct4. Osteopontin, a marker protein of hard tissues, was expressed predominantly in the nonwovens populated with cocultures (**I**,**L**). The periodontium-related marker periostin was found mainly in the cocultures (**O**,**R**) or PDLFs (**N**,**Q**), but barely in hMSCs (**M**,**P**). The white arrows in (**A**,**B**,**D**,**F**) exemplarily indicate positively stained cells, which are otherwise hard to recognize. Scale bars represent 100 µm.

Oct4 is a so-called “stem cell marker” protein expressed in the nuclei of cells with high developmental potency, including hMSCs [76]. Osteopontin is usually found in hard tissues and is also known as bone sialoprotein 1 (BSP-1). It is located in the extracellular matrix and functions as a linker protein and chelator for inorganic cations, thereby inhibiting tissue mineralization [77,78]. Periostin is also a component of the extracellular matrix and is associated with mesenchymal/mesodermal tissues, such as the periodontal ligament. It is a ligand for cellular integrin receptors [79]. These three proteins were visualized in eGHA and eGHA_ap_ nonwovens populated with either hMSCs, PDLFs, or cocultures via immunohistochemistry after a culture period of 21 d. With the help of these characteristic biomarkers, cell types could be distinguished, which was especially important in the coculture experiments. Additionally, the mesenchymal intermediate filament protein vimentin was immunodecorated in all samples to visualize all cells, since both hMSCs and PDLFs express this protein. Hematoxylin counterstaining was performed to enhance the contrast. The results of these experiments are depicted in Figure 6. The biomarker expression was assessed visually and only qualitatively.

Oct4 was expressed in hMSCs on both eGHA (Figure 6D) and eGHA_ap_ (Figure 6A). The nuclear localization of Oct4 is best seen in the inset in Figure 6D, where green vimentin staining is also visible. PDLFs (Figure 6B,E) barely expressed Oct4, and only some cells, presumably hMSCs, in the coculture setting (Figure 6C,F) stained positive for this protein.

Osteopontin could be detected in eGHA and eGHA_ap_ nonwovens populated with the cocultures (Figure 6I,L) and to a lesser extent on PDLF- and hMSC-harboring scaffolds (Figure 6H,K and Figure 6G,J, respectively).

The mesodermal/mesenchymal marker periostin was expressed predominantly on nonwovens harboring the cocultures (Figure 6O,R) and those cultivating only PDLFs (Figure 6N,Q). Notably, hMSCs did barely express periostin (Figure 6M,P).

From the qualitative assessment of the immunohistochemical stainings, there was no apparent difference between eGHA and eGHA_ap_ scaffolds. Nonetheless, the applied culture methods and the gelatin/hydroxyapatite scaffolds allowed differential expression of relevant biomarker proteins in hMSCs, PDLFs, and cocultures of both cell types. The biochemical and biomechanical properties of the nonwovens either actively supported or passively permitted the differentiation or stemness maintenance of these periodontal cell types. Biomarker expression, especially periostin and osteopontin, was enhanced in interactive cocultures of hMSCs and PDLFs, indicating more efficient cell differentiation than in monocultures of each cell type. Thus, hMSCs are supposed to support the PDLFs´ differentiation on the nonwovens irrespective of the presence of additional porosity.

## 4. Discussion

Periodontal tissue engineering remains a major challenge in dental medicine [80,81]. Because of the complex histological architecture and the multiple functions of this anatomic area, tissue engineering approaches to treat periodontal defects need to consider many biochemical and biomechanical parameters [82]. Current periodontal regeneration strategies, including guided tissue or bone regeneration, still have major shortcomings, such as the selection of the optimal biomaterial [83]. Optimization of bone regeneration in terms of biodegradation, rigidity, and stability is also complex and time-consuming [84]. Surface modifications of tissue engineering scaffolds differentially influence the behavior of periodontal cells, i.e., parameters such as proliferation and differentiation, which makes it difficult to spatiotemporally control proper tissue formation [85,86]. Therefore, innovative novel biomaterial-based approaches that overcome current shortcomings are needed in the field of regenerative dentistry. In this context, we aimed at generating in situ cross-linked gelatin/hydroxyapatite nonwovens for periodontal tissue engineering that enabled adhesion, proliferation, and differentiation of two different periodontal cell types, i.e., hMSCs and PDLFs. Their overall suitability for this purpose was evaluated in vitro.

Gelatin was chosen as the basic material because it is a main component of periodontal soft tissues [87]. It possesses innate arginine-glycine-aspartate (RGD) amino acid motifs, which enable interactions with cellular integrins [88]. This facilitates cellular adhesion and differentiation and supports cellular mechanotransduction, a process by which extracellular biomechanical signals such as the stiffness (Young´s modulus) of the pericellular environment are transformed into intracellular biochemical signals [89]. As mechanotransduction is a key factor in determining cell behavior, fine-tuning of biomaterials´ mechanical properties can significantly enhance the regenerative capacity and success of tissue engineering approaches [5,90,91,92]. Additionally, gelatin reduces inflammatory responses in in vivo applications, since it only moderately triggers the immune system [93,94]. In the context of periodontal hard tissue regeneration, biomineralized gelatin/collagen containing intrafibrillar calcium phosphate mineral also favors osteogenic differentiation of periodontal stem cells [95]. This is a highly desirable property, since the soft tissue/hard tissue interaction, i.e., the periodontal ligament/alveolar bone interface, is key to the anchoring of teeth [96].

In the present study, additionally to gelatin, hydroxyapatite was incorporated into the nonwovens. Hydroxyapatite is the main inorganic component of periodontal hard tissues, i.e., cementum and the alveolar bone. This mineralized addition generally facilitates bone regeneration and influences the differentiation of cells with osteogenic potential, e.g., PDLFs or hMSCs [97,98,99,100,101,102,103]. Exemplarily, Inanç and colleagues reported that PDLFs cultured in osteogenic medium within chitosan–hydroxyapatite microspheres showed convincing signs of osteogenic differentiation [98]. Thus, the combination of collagen and hydroxyapatite in our approach combined the favorable properties of both materials and therefore appeared optimal for applications in periodontal tissue engineering.

Incorporation of additional porosity was aimed at increasing the porosity of some of the model surfaces. There were two major reasons for this: (i) it increases the permeability of the material to allow for efficient penetration of nutrients and oxygen [104]; (ii) it increases the adhesion surface for the cells [105]. Porous tissue engineering materials have been proven suitable for tissue regeneration, as exemplified by efficient bone regeneration [106,107]. They have also been investigated in the context of periodontal tissue engineering and were shown to enhance angiogenesis in the periodontal ligament [108]. A chitosan-based approach with a porous, trilayered biomaterial that imitated bone, gingiva, and the periodontal ligament supported the growth of GFs, osteoblasts, and PDLFs [109]. This proved the overall applicability of porous biomaterial scaffolds for periodontal tissue engineering. The potential advantages of additional porosity in a nonwoven populated with hMSCs, PDLFs, or cocultures of both cell types, however, have never been investigated directly. The simultaneous testing of eGHA and eGHA_ap_ nonwovens with the two cell types was therefore expected to uncover potential effects of the increased porosity on cell adhesion, proliferation, and differentiation (see below).

Electrospinning is a widely used method for fabricating biomaterials for tissue engineering and has been applied to many subdisciplines of regenerative medicine. It enables fast, efficient, and reproducible generation of nonwovens composed of natural, synthetic, or semisynthetic polymers [110]. By systematically modifying electrospinning parameters, fibers can be tailored to the specific experimental or clinical needs. Upscaling to industrial fabrication levels is also possible, making the technique feasible for bench-to-bedside translation of tissue engineering innovations [111]. However, exact determination of pore structure and size is difficult in electrospinning, since it varies with fiber diameter [112]. Exemplarily, alternative current biomaterial fabrication methods include 3D bioprinting or melt spinning [113,114,115,116]. While oral applications of melt spinning have been scarcely reported in the literature, 3D bioprinting has been successfully applied to periodontal ligament cells [117]. As an example, a gelatin methacryloyl hydrogel was produced in a microextrusion approach as a cell-laden scaffold for periodontal tissue engineering [118]. Despite the promising features of 3D bioprinting, however, it has some drawbacks, including the need for specific bioinks and inaccuracies in droplet placement [119]. In comparison with 3D bioprinting, where cells are possibly directly incorporated into the scaffold, the population of electrospun biomaterials by externally seeded cells allows for the selective study of cellular penetration. The latter point is important when it comes to the ingrowth of cells from wound margins in periodontal defects in vivo. Because of these reasons, we chose to use electrospinning for the fabrication of the presented nonwovens.

As indicated above, cellular mechanotransduction is a main determinant of cellular behavior [90]. Among other factors, the elasticity/stiffness, i.e., the elastic/Young´s modulus, of the extracellular environment strongly influences cells’ response to mechanical stimuli. Thus, biomaterials for tissue regeneration purposes need to be characterized mechanically [120,121]. Interestingly, the stiffness of a biomaterial can provide all of the necessary biological information to induce proliferation and differentiation of certain cell types. Similar materials are often designated as “cell-instructive” [122,123]. In the periodontal context, this is best exemplified by epitheliogenesis of the gingival epithelium. It was thought for a long time that only cocultures of GFs and GKs led to proper stratification and differentiation of GKs, i.e., that GFs were needed to induce gingival epitheliogenesis. However, it was recently shown that a nanofibered gelatin-based nonwoven could induce epitheliogenesis of GKs by its stiffness alone, which was in the low kPa range [30]. Numerical values for the elastic modulus of the human periodontal ligament have varied considerably in the literature depending on the methodology used for its determination. While some authors have proposed nonlinear mechanical behavior of the ligament, others have described values in the MPa range [124,125]. The values determined for the eGHA and eGHA_ap_ scaffolds were in the low kPa range and thus clearly lower. Nonetheless, both scaffolds enabled adhesion, survival, and typical biomarker expression of hMSCs, PDLFs, and cocultures of the cell types. Since the nonwovens were used only for a proof-of-principle study under static in vitro conditions, this notable difference in elastic moduli was not necessarily relevant. However, this point needs to be considered for potential later in vivo application for two main reasons: (i) the material needs to possess a sufficient dimensional stability for (surgical) use in the periodontium; (ii) as molecular cell responses rely on mechanical cues, proper cellular differentiation/lineage determination or the maintenance of the cellular phenotype might strongly depend on a certain extracellular stiffness. Therefore, when adapting the principle of the presented nonwovens to in vivo applications, the Young´s modulus might need an appropriate adjustment.

As shown by the SEM analysis, eGHA and eGHA_ap_ scaffolds were efficiently populated by both hMSCs and PDLFs. Interestingly, cellular morphologies exhibited no obvious abnormalities, which indirectly showed that the nonwovens supported proper morphogenesis of both cell types. Simultaneous population by cocultures was also possible, thereby proving that the biomaterial contained all of the necessary biomechanical information for the adhesion and growth of these periodontal cell types. The comparison of the SEM micrographs between d 10 and d 21 also supported the notion that the cells could proliferate on the scaffolds, since cell densities increased according to visual assessment. Although SEM is valuable in the study of cell shape and the geometry of the biomaterial itself, quantitative assessment of cell densities is difficult for several reasons. First, cell margins cannot be differentiated with sufficient certainty (see, e.g., Figure 2C,D). Second, cells penetrating the scaffolds, i.e., cells not lying directly at the surface of the scaffold, cannot be visualized reliably. Consequently, DAPI staining was applied to stain cross-sections of cell-populated eGHA and eGHA_ap_ nonwovens to quantitatively determine cell densities. Three main questions were relevant in this context: (i) how did each cell type perform on each scaffold? (ii) what influence did the type of scaffold have on the cell densities? (iii) what were the temporal dynamics of cell growth on each platform? Concerning (i), it was found that PDLFs and cocultures exhibited higher cell densities on the nonwovens. Regarding the latter finding, it must be considered that PDLFs and hMSCs cannot be distinguished via DAPI staining, meaning that the calculated cell densities for the coculture setting represented average values for both cell types. The relatively low cell densities for hMSC-populated scaffolds might be caused by either insufficient adhesion or slow proliferation of the cells on the scaffold. Regarding question (ii), cell densities were generally higher on eGHA_ap_ than on eGHA. This effect was independent of the cell type and point in time, meaning that the additional porosity was responsible for this finding. This indicates that the additional porosity either favored cellular adhesion or cell survival or promoted proliferation. The answer to question (iii) contributed to the solution of this problem. Since there was a trend for increasing cell densities over time for PDLFs and the cocultures but not for hMSCs alone, it can be assumed that the additional porosity mainly favored cellular adhesion or survival but did not directly support proliferation (at least not for hMSCs). Otherwise, one would have expected increasing cell numbers for all cell types on eGHA_ap_, or at least no difference between eGHA and eGHA_ap_ cell densities for hMSCs. Thus, it can be concluded that the additional porosity was beneficial in terms of initially allowing hMSC and PDLF colonization of the eGHA_ap_ scaffolds, while the conditions for proliferation were more suitable for PDLFs. In the coculture setting, high cell densities were supposed to be best explained by a predominance of PDLFs or a synergistic interaction of both cell types. This question remains to be answered with other experimental approaches. Interestingly, high initial seeding densities have been shown to be advantageous in tissue engineering applications, as exemplified by a poly-glycolic acid nonwoven designed for temporomandibular joint regeneration [124]. It can thus be speculated that a biomaterial that allows for high cell densities in the initial healing phase might outperform alternative approaches in the long run. This will be an interesting point to examine in the future.

Cellular density is, however, not the only parameter to be considered in the evaluation of biomaterials for periodontal tissue engineering. If a biomaterial is intended to be used as a guiding structure for tissue regeneration, colonization by tissue-resident cells in vivo is important for the restitution of the tissue. This enables the spatiotemporally defined degradation of the scaffold and the subsequent replacement thereof by native tissue [126]. Three-dimensional cellular interactions allow more efficient regeneration [127]. Therefore, the maximum cell penetration into the scaffolds was assessed next. Notably, the trends seen for the cell densities were not mirrored by those for the cell penetration. As described in detail in the Results section, there was no clear advantage for eGHA_ap_ scaffolds for maximum cell penetration. This indicates that the additional porosity did not necessarily favor cell migration into the nonwovens. There was only a slight trend for higher penetration depths for PDLFs and the coculture setting and a tendency towards higher penetration depths over time. Detailed analysis of cellular migration behavior in biomaterials is complex [128,129]. However, it is evident that the exact nanotopography of a biomaterial strongly influences the capability of the cells to invade the material. Material parameters, such as the density of cell adhesion points and the width of the pores, are key in determining cell migration [130]. Cellular properties, including deformability and the secretion of proteolytic enzymes, also play a role in this process [131]. Although some studies have addressed the question of how to enhance cellular penetration into nonwoven scaffolds, it is still unclear what therapeutic effect results from these efforts [132,133,134]. Thus, detailed, comparative in vivo studies on these issues are needed in the periodontal context. It is of great importance to discover whether higher cell densities and/or higher cell penetration depths are beneficial for periodontal tissue engineering in vivo. In summary, additional porosity supported higher cell densities but did not unambiguously boost penetration of hMSCs or PDLFs into the nonwovens. Compared with eGHA nonwovens, eGHA_ap_ nonwovens showed lower dimensional stability (see Figure 1). This is unfavorable for a potential surgical application [82]. Therefore, the potential biological benefits of the additional porosity must additionally be traded off against clinical handling.

Another important criterion for evaluating the suitability of the nonwovens for later in vivo application for periodontal tissue engineering is the metabolic integrity/viability of the cells. The latter can be assessed by different assays, e.g., Alamar Blue^®^ staining, which quantifies the reductive capacity of cells and thereby indirectly investigates mitochondrial function and thus cell viability [135]. When analyzing the viability/metabolic activity of cells over time, insights into the proliferative behavior are additionally gained [136]. As with the cell densities, the metabolic activity of hMSCs, PDLFs, and the cocultures was higher on eGHA_ap_ than on eGHA nonwovens. Especially for PDLFs and the cocultures, but also for hMSCs, on eGHA_ap_, there was additionally a trend towards higher metabolic activity over time. While the findings for the PDLFs and the cocultures underscored the suspected efficient proliferation of PDLFs on the nonwovens, the results for the hMSCs on eGHA_ap_ with increasing metabolic activity over time were not in accordance with the data on nearly constant cell densities (see above). The reasons for this, however, remain elusive.

Finally, the expression of characteristic biomarker proteins was assessed in hMSCs, PDLFs, and cocultures of both cell types on eGHA and eGHA_ap_ nonwovens. hMSCs expressed Oct4, a stem cell marker, as expected. Oct4 is a key transcription factor in maintaining stemness properties of cells with a great developmental potency [137]. Since hMSCs on both eGHA and eGHA_ap_ scaffolds expressed Oct4, it can be assumed that the nonwovens sustained hMSCs stemness properties over a period of at least three weeks. This is a desirable feature, since it allows for enhancement of the regenerative potential at defect sites in vivo. The transfer of stem cells and subsequent targeted, spatiotemporal differentiation of the cells at a periodontal defect site may be possible in the future [138,139]. Osteopontin was expressed mainly under the coculture conditions and to some extent in the PDLFs and hMSCs. Osteopontin expression in hMSCs and PDLFs may be interpreted as a first step in osteoblastic differentiation of the cells [140,141,142,143]. The comparatively high expression of osteopontin in the cocultures was, however, especially interesting. This finding is the first hint that the cocultures were interactive, i.e., mutually influenced each other. It is tempting to speculate that the coculture of hMSCs and PDLFs might enhance hard tissue formation in the periodontium through, e.g., soluble factors. Since it has been described in the literature that hMSCs interact with various periodontal cell types, including GFs and osteoblasts, it is plausible that such mechanisms also play a role in determining osteogenic differentiation of PDLFs [74,75]. PDLFs show an inherent osteogenic potential, which underscores the plausibility of the hypothesis [85,144]. Such coculture approaches thus enhance the therapeutic potential in the context of alveolar bone loss during periodontitis. This is important, since periodontal soft tissues such as the junctional epithelium regenerate much faster than the periodontal hard tissues. By enhancing hard tissue regeneration with the help of coculture approaches, proper restitution of the alveolar compartment is more likely [145]. Periostin expression, a marker of mesodermal cells such as PDLFs, was also most prominently expressed in the cocultures and PDLFs, underscoring that soft tissue marker proteins were sustained in the mono- and coculture setting on the nonwovens. Taken together, the immunohistochemical analysis of the biomarker expression did not unambiguously reveal a difference between eGHA and eGHA_ap_ nonwovens but emphasized the suitability of the nonwovens as appropriate scaffolds for periodontal tissue engineering, enabling interactions of hMSCs and PDLFs.

## 5. Conclusions

We herein present new electrospun gelatin/hydroxyapatite-based nonwovens for periodontal tissue engineering. They proved suitable for the adhesion, survival, and biomarker expression of hMSCs, PDLFs, and coculture of both cell types. Incorporation of additional porosity into a subset of the nonwovens revealed that increased porosity favored higher cell densities and metabolic activities of the cells. However, additional porosity did not lead to more efficient migration of cells into the scaffolds. Upon examination of differentiation biomarkers, hMSCs maintained stemness properties, as exemplified by Oct4 expression. Interestingly, cocultures of PDLFs and hMSCs showed enhanced expression of osteopontin when compared with both monoculture conditions. This indicates that the cocultures were actually interactive, i.e., revealed a mutual influence of both cell types. Thus, the scaffolds allowed for cellular interactions through yet unknown mechanisms. Taken together, the characteristics shown herein render the novel biomaterials promising candidates for prospective periodontal tissue regeneration.

## Data Availability

Not applicable.

## Supplementary Materials

The following supporting information can be downloaded at: https://www.mdpi.com/article/10.3390/pharmaceutics14061286/s1, Figure S1: Typical, idealized load–indentation curve of a Bioindenter™ experiment, Figure S2: Exemplary load–indentation curve of an eGHA scaffold, Figure S3: Exemplary load–indentation curve of an eGHA_ap_ scaffold, Figure S4: Scanning electron micrographs of eGHA/eGHA_ap_ scaffolds after 10 d. Table S1: Quantitative assessment of the cell densities (cells/µm^2^) on eGHA and eGHA_ap_ nonwovens populated with hMSCs, PDLFs, and cocultures for 3, 7, 10, 14, and 21 d, Table S2: Quantitative assessment of the cell penetration depths (µm) on eGHA and eGHA_ap_ nonwovens populated with hMSCs, PDLFs, and cocultures for 3, 7, 10, 14, and 21 d, Table S3: Quantitative assessment of the mean cellular metabolic activity (%) on eGHA and eGHA_ap_ nonwovens populated with hMSCs, PDLFs, cocultures, and no cells (empty) for 1, 3, 7, 10, 14, and 21 d.
