# Supplementary material for: Novel In Situ-Cross-Linked Electrospun Gelatin/Hydroxyapatite Nonwoven Scaffolds Prove Suitable for Periodontal Tissue Engineering
Source: Pharmaceutics. 2022 Jun 16;14(6):1286. doi: 10.3390/pharmaceutics14061286 (PMC9230656; doi:10.3390/pharmaceutics14061286)
Supplement: Supplementary file 1 [file pharmaceutics-14-01286-s001.zip › pharmaceutics-1756197-supplementary.pdf]

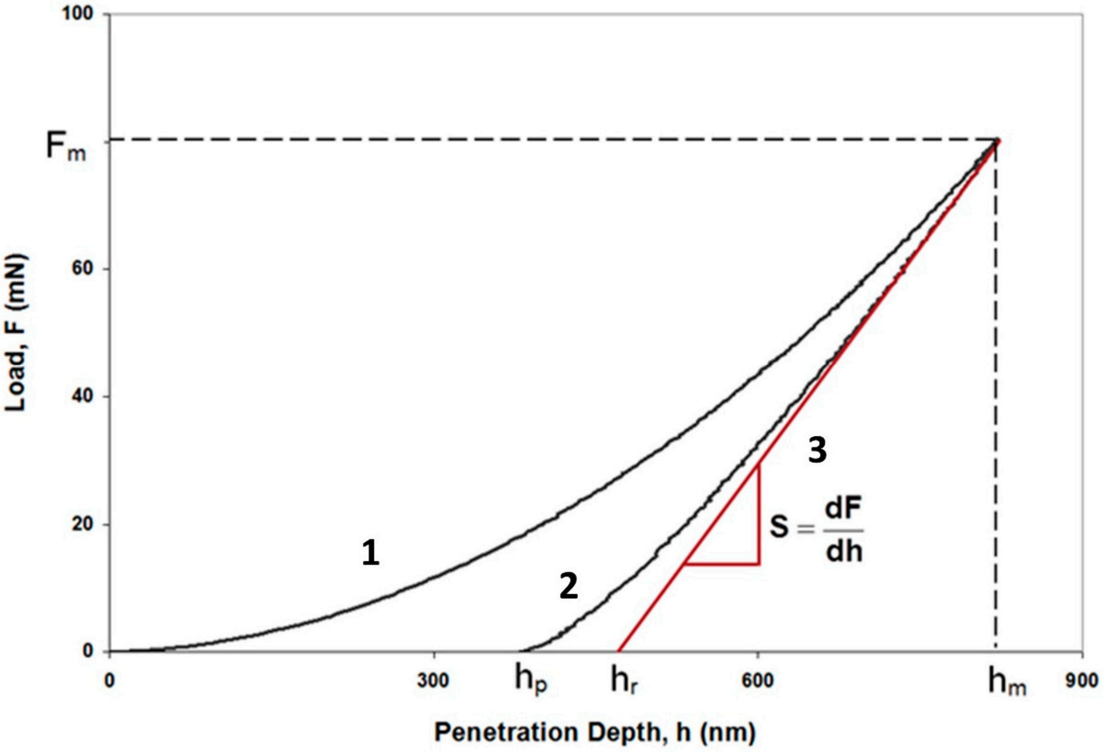

Figure S1. Typical, idealized load–indentation curve of a Bioindenter™ experiment. (1) describes the loading curve; (2), the unloading curve. According to the method described by Oliver and Pharr, the Young´s modulus can be calculated from values of the load–indentation curve and the geometric parameters of the indenter [76]. The tangent (3) at the point of the maximum indentation depth (h_m) is used to calculate the contact stiffness S (see Equation (1)). F_m is the maximum force during the experiment, yielding h_m. h_p describes the permanent indentation depth h after omission of the load F. h_r is the theoretical intersection between the tangent (3) and the *x*-axis.

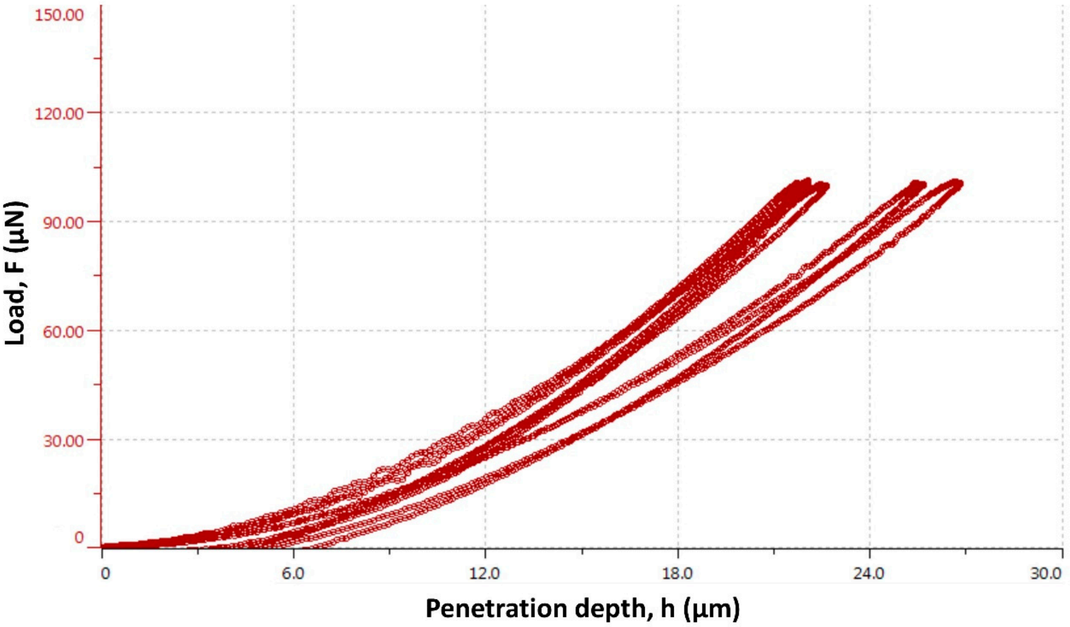

Figure S2. Exemplary load–indentation curve of an eGHA scaffold. The scaffold was prepared as described in the Material and Methods section. The graph shows the load–indentation curves of five subsequent measurements of the same sample. The load F is plotted on the *y*-axis, while the penetration depth h is plotted on the *x*-axis. For this scaffold, the calculated Young´s modulus was 6.41 ± 0.59 kPa.

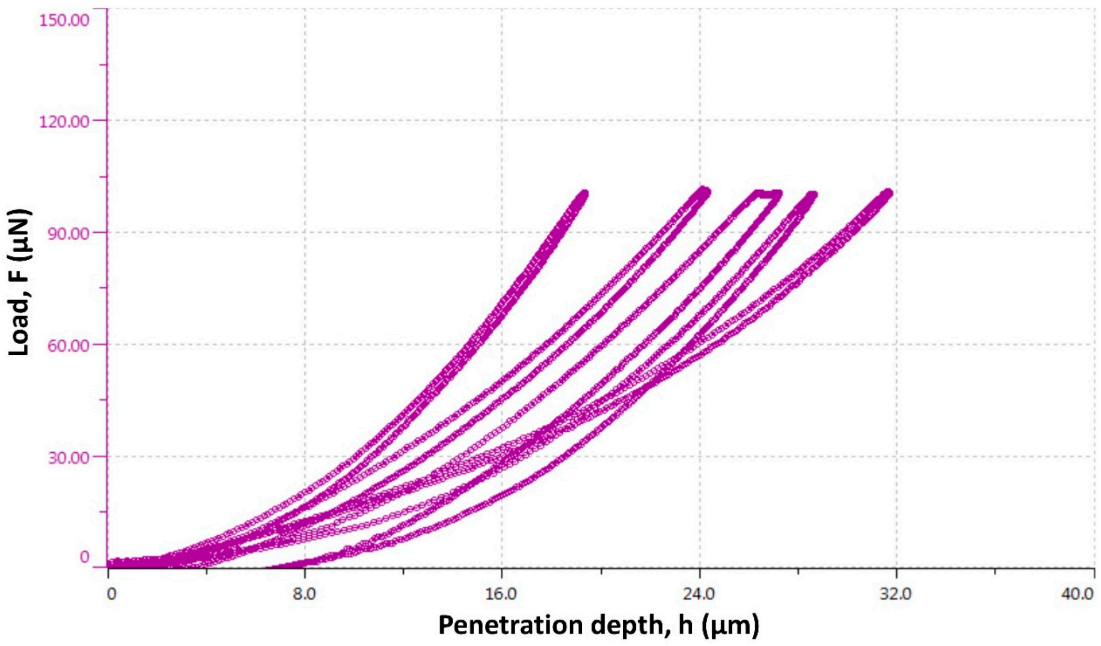

Figure S3. Exemplary load–indentation curve of an eGHA$_{ap}$ scaffold. The scaffold was prepared as described in the Material and Methods section. The graph shows the load–indentation curves of five subsequent measurements of the same sample. The load F is plotted on the *y*-axis, while the penetration depth h is plotted on the *x*-axis. For this scaffold, the calculated Young´s modulus was 6.45 ± 0.97 kPa.

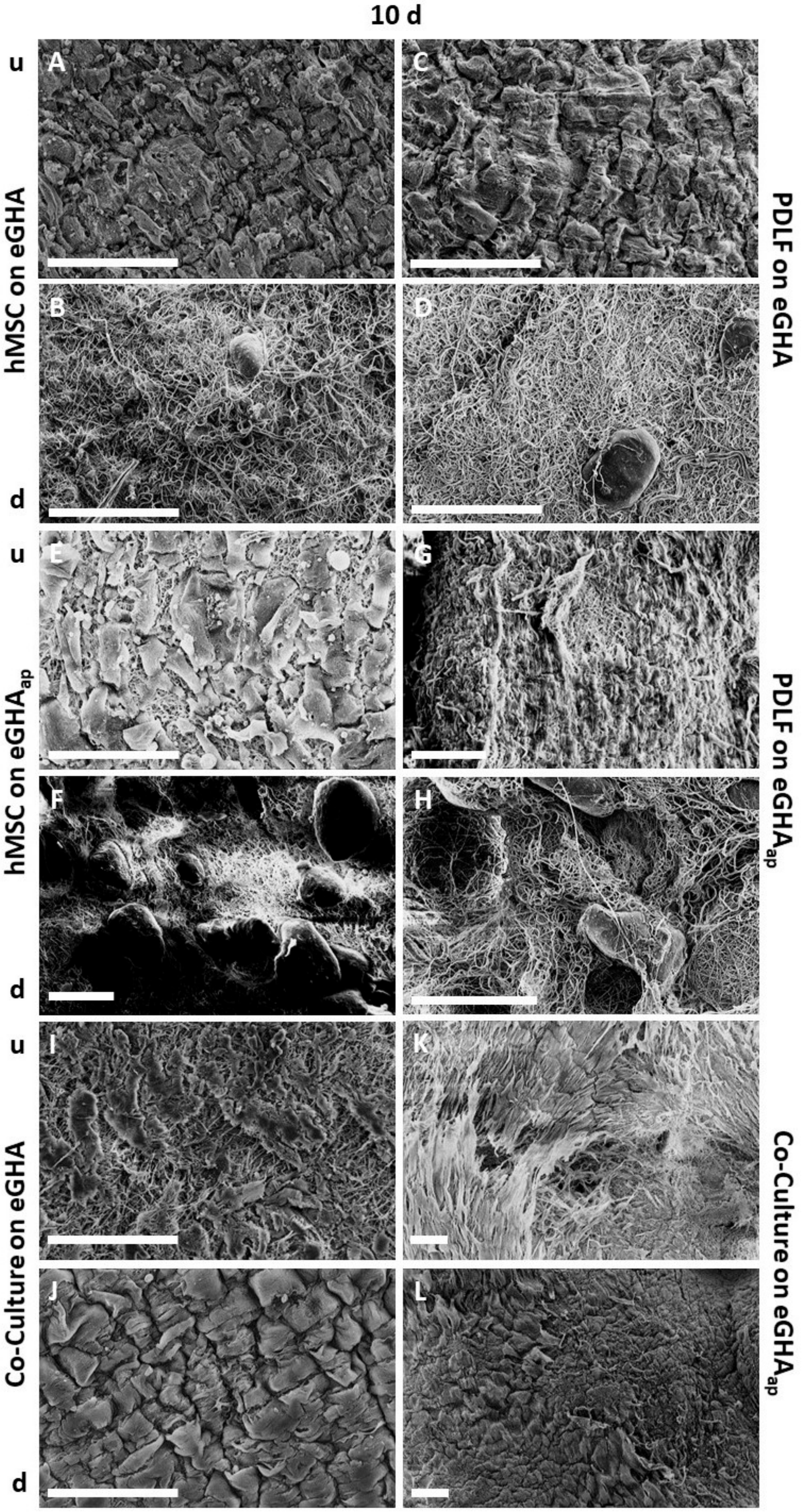
10 d
u
d
u
d
u
d
A
B
C
D
E
F
G
H
I
J
K
L
hMSC on eGHA
PDLF on eGHA
hMSC on eGHA$_{ap}$
PDLF on eGHA$_{ap}$
Co-Culture on eGHA
Co-Culture on eGHA$_{ap}$

Figure S4. Scanning electron micrographs of eGHA/eGHA$_{ap}$ scaffolds after 10 d. hMSCs (A,B,E,F), PDLFs (C,D,G,H), and cocultures of both (I–L) were seeded on either eGHA (A–D,I,J) or eGHA$_{ap}$ scaffolds (E–H,K,L) and prepared for scanning electron microscopy (SEM) analysis after 10 d. The upsides (u) of the monocultures (A,C,E,G) were densely populated with either hMSCs or PDLFs, while the downsides (d) (B,D,F,H) illustrated the geometric configurations of the nonwovens. In the cocultures, (u) were populated by hMSCs (I,K) and (d) with PDLFs (J,L). Details are given in the main text. All scaffolds, irrespective of the presence of additional porosity, were densely covered with the indicated cells, proving the overall suitability of the eGHA/eGHA$_{ap}$ nonwovens for the adhesion and spreading of periodontal cells. The cell morphologies could be described as polygonal or spindle-like. Scale bars represent 100 μm.

Table S1. Quantitative assessment of the cell densities (cells/μm^2) on eGHA and eGHA$_{ap}$ nonwovens populated with hMSCs, PDLFs, and cocultures for 3, 7, 10, 14, and 21 d. The number (N) of evaluated samples per condition is indicated in the fourth column. The median (fifth column) and mean calculated cell densities (sixth column) and the corresponding standard deviations (SD, seventh column) are also depicted.

			N	Median (cells/μm^2)	Mean (cells/μm^2)	SD (cells/μm^2)
**hMSC**	**3 d**	**eGHA**	6	0.0098372	0.008729	0.0019555
		**eGHA$_{ap}$**	7	0.0731069	0.0729291	0.049423
	**7 d**	**eGHA**	2	0.0026622	0.0026622	0.0015154
		**eGHA$_{ap}$**	6	0.0658464	0.0777959	0.0398618
	**10 d**	**eGHA**	5	0.02066	0.0258829	0.0117035
		**eGHA$_{ap}$**	2	0.0439267	0.0439267	0.0142013
	**14 d**	**eGHA**	7	0.0250599	0.0371012	0.0331185
		**eGHA$_{ap}$**	4	0.0205074	0.0222475	0.0157218
	**21 d**	**eGHA**	7	0.0296688	0.0300406	0.0137333
		**eGHA$_{ap}$**	5	0.044212	0.0419517	0.0209356
**PDLF**	**3 d**	**eGHA**	not evaluable	not evaluable	not evaluable	not evaluable
		**eGHA$_{ap}$**	7	0.0573319	0.0621296	0.0263405
	**7 d**	**eGHA**	5	0.0051102	0.0059401	0.0039619
		**eGHA$_{ap}$**	7	0.1630009	0.1685361	0.0269491
	**10 d**	**eGHA**	5	0.0596111	0.06095	0.0151834
		**eGHA$_{ap}$**	5	0.394736	0.2959878	0.1899214
	**14 d**	**eGHA**	3	0.0997332	0.1115	0.0280429
		**eGHA$_{ap}$**	5	0.1330316	0.155904	0.0529579
	**21 d**	**eGHA**	10	0.0883228	0.0943171	0.0376992
		**eGHA$_{ap}$**	5	0.3205851	0.3225946	0.2132816
**Coculture**	**3 d**	**eGHA**	5	0.0070459	0.0066426	0.0016389
		**eGHA$_{ap}$**	6	0.008123	0.0084105	0.003103
	**7 d**	**eGHA**	6	0.0222034	0.0463756	0.0422479
		**eGHA$_{ap}$**	10	0.0931795	0.1105075	0.042763
	**10 d**	**eGHA**	4	0.0492225	0.0511814	0.0121283
		**eGHA$_{ap}$**	4	0.0449748	0.070117	0.0514661
	**14 d**	**eGHA**	8	0.1026672	0.0839942	0.0500624
		**eGHA$_{ap}$**	10	0.1524226	0.1753802	0.0486237
	**21 d**	**eGHA**	9	0.0899736	0.117514	0.1117002
		**eGHA$_{ap}$**	9	0.1108366	0.2001065	0.1171855

Table S2. Quantitative assessment of the cell penetration depths (µm) on eGHA and eGHA$_{ap}$ nonwovens populated with hMSCs, PDLFs, and cocultures for 3, 7, 10, 14, and 21 d. The number (N) of evaluated samples per condition is indicated in the fourth column. The median (fifth column) and mean calculated cell penetration depths (sixth column) and the corresponding standard deviations (SD, seventh column) are also depicted.

			N	Median (µm)	Mean (µm)	SD (µm)
**hMSC**	**3 d**	**eGHA**	6	85.25728	86.29339	32.91673
		**eGHA$_{ap}$**	7	79.0406	95.02634	33.01124
	**7 d**	**eGHA**	2	136.3228	136.3228	98.59273
		**eGHA$_{ap}$**	6	129.6621	151.2725	60.20134
	**10 d**	**eGHA**	5	658.9677	580.9928	223.7223
		**eGHA$_{ap}$**	2	164.7419	164.7419	53.37823
	**14 d**	**eGHA**	7	201.5979	212.6357	89.83667
		**eGHA$_{ap}$**	4	153.6407	153.8627	33.84394
	**21**	**eGHA**	7	544.4033	500.2522	199.0847
		**eGHA$_{ap}$**	5	258.4361	208.7027	96.8738
**PDLF**	**3 d**	**eGHA**	not evaluable	not evaluable	not evaluable	not evaluable
		**eGHA$_{ap}$**	7	39.96435	41.61367	12.98595
	**7 d**	**eGHA**	5	31.97148	37.30006	24.42669
		**eGHA$_{ap}$**	7	208.7027	209.8446	56.68004
	**10 d**	**eGHA**	5	288.6314	280.4609	202.6529
		**eGHA$_{ap}$**	5	332.1482	353.9953	93.2345
	**14 d**	**eGHA**	3	143.8717	150.3844	40.36039
		**eGHA$_{ap}$**	5	283.3028	302.3081	45.58195
	**21**	**eGHA**	10	464.4746	378.862	201.6091
		**eGHA$_{ap}$**	5	422.734	410.4783	173.4899
**Coculture**	**3 d**	**eGHA**	3	161.6336	190.0527	67.68788
		**eGHA$_{ap}$**	5	41.74054	44.22721	21.98286
	**7 d**	**eGHA**	6	133.2145	169.1824	79.15876
		**eGHA$_{ap}$**	10	201.1539	204.4399	68.58984
	**10 d**	**eGHA**	4	342.3613	313.9422	133.6411
		**eGHA$_{ap}$**	4	193.161	189.6086	55.56817
	**14 d**	**eGHA**	8	243.3385	235.7897	25.48875
		**eGHA$_{ap}$**	10	301.5088	322.6455	81.27155
	**21**	**eGHA**	9	340.141	308.0709	136.0314
		**eGHA$_{ap}$**	9	247.779	234.2602	37.66508

			N	Mean [%]	SD [%]
1 d	empty	eGHA	9	5.018161	1.218843
		$eGHA_{ap}$	9	3.429422	0.6687911
	hMSC	eGHA	15	20.23757	3.064675
		$eGHA_{ap}$	15	26.39089	3.391284
	PDLF	eGHA	15	20.55165	4.687862
		$eGHA_{ap}$	15	25.29921	6.680117
	Co-Culture	eGHA	18	30.90285	7.913007
		$eGHA_{ap}$	18	37.18791	10.79863
3 d	empty	eGHA	9	5.407166	0.881299
		$eGHA_{ap}$	9	3.618524	0.6053672
	hMSC	eGHA	15	30.57237	20.84409
		$eGHA_{ap}$	15	25.60262	5.503864
	PDLF	eGHA	15	31.43811	4.762844
		$eGHA_{ap}$	15	38.09538	11.41298
	Co-Culture	eGHA	18	42.51755	6.113959
		$eGHA_{ap}$	18	45.54092	10.46634
7 d	empty	eGHA	9	5.651971	1.861113
		$eGHA_{ap}$	9	3.888108	1.713384
	hMSC	eGHA	15	25.18601	7.43302
		$eGHA_{ap}$	15	33.59624	6.677269
	PDLF	eGHA	15	43.76426	6.762997
		$eGHA_{ap}$	15	62.66017	10.46475
	Co-Culture	eGHA	18	51.00284	9.842653
		$eGHA_{ap}$	18	63.55691	6.317564
10 d	empty	eGHA	9	4.913745	0.9530044
		$eGHA_{ap}$	9	2.668842	0.9634505
	hMSC	eGHA	15	28.06508	7.184959
		$eGHA_{ap}$	15	36.13512	8.734512
	PDLF	eGHA	15	58.71049	3.33624
		$eGHA_{ap}$	15	69.78634	9.768611
	Co-Culture	eGHA	18	54.42184	6.433946
		$eGHA_{ap}$	18	71.92907	8.600621
14 d	empty	eGHA	9	4.187012	0.6427488
		$eGHA_{ap}$	9	2.271207	0.4109346
	hMSC	eGHA	15	20.32461	13.60807
		$eGHA_{ap}$	15	36.47787	5.438348
	PDLF	eGHA	15	56.31027	10.83526
		$eGHA_{ap}$	15	68.51553	6.292524
	Co-Culture	eGHA	18	55.57401	9.230831
		$eGHA_{ap}$	18	64.31733	5.458969
21 d	empty	eGHA	9	3.93965	0.9652498
		$eGHA_{ap}$	9	2.299034	0.4017685
	hMSC	eGHA	15	23.55676	16.74048
		$eGHA_{ap}$	15	46.13785	6.420403
	PDLF	eGHA	15	64.11949	13.56675
		$eGHA_{ap}$	15	67.5882	7.637498
	Co-Culture	eGHA	18	56.95062	9.03093
		$eGHA_{ap}$	18	67.31662	7.241198

Table S3. Quantitative assessment of the mean cellular metabolic activity (%) on eGHA and $eGHA_{ap}$ nonwovens populated with hMSCs, PDLFs, cocultures, and no cells (empty) for 1, 3, 7, 10, 14, and 21 d. The number (N) of evaluated samples per condition is indicated in the fourth column. The mean calculated cellular metabolic activities (fifth column) and the corresponding standard deviations (SD, sixth column) are also depicted.
